# Saroglitazar Facilitates Insulin De-escalation and Improves Metabolic Parameters in Pioglitazone-Ineligible Non-metabolic Dysfunction-Associated Steatotic Liver Disease (MASLD) Type 2 Diabetes: Real-World Evidence From India

**DOI:** 10.7759/cureus.117669

**Published:** 2026-10-09

**Authors:** Indumathi Kuberan

**Affiliations:** 1 Diabetes and Endocrinology, Indu’s Diabetes Clinics, Mumbai, IND

**Keywords:** insulin sensitizer, peroxisome proliferator-activated receptor (ppar)-α/γ agonist, pioglitazone contraindication, saroglitazar, type 2 diabetes mellitus

## Abstract

Introduction: Type 2 diabetes mellitus (T2DM) is a major global health burden, particularly in India. Pioglitazone is an established thiazolidinedione insulin sensitizer. It is contraindicated in heart failure and is clinically unsuitable for many patients with edema, osteoporosis, or bladder cancer risk, creating a therapeutic gap. Saroglitazar, a dual peroxisome proliferator-activated receptor (PPAR)-α/γ agonist approved in India for T2DM, diabetic dyslipidemia, and metabolic dysfunction-associated steatotic liver disease (MASLD), has insulin-sensitizing properties, but evidence in non-MASLD T2DM patients remains limited.

Methods: This single-center, retrospective, real-world study analyzed the medical records of adults with T2DM who were deemed ineligible for pioglitazone and were initiated on saroglitazar 4 mg once daily as add-on therapy between June 2025 and March 2026. All consecutive eligible patients with documented follow-up at approximately 12 and 24 weeks were included (n = 60). Data on demographics, comorbidities, concomitant antidiabetic therapy and its modifications, glycated hemoglobin (HbA1c), fasting and postprandial blood glucose (FBG, PPBG), lipid profile, liver enzymes, serum creatinine, body weight, and adverse events were extracted from outpatient case records, electronic medical records, and laboratory reports using a standardized data collection form. Changes from baseline to 12 and 24 weeks were assessed using two-tailed paired t-tests with 95% confidence intervals (CIs); P < 0.05 was considered statistically significant.

Results: Mean age was 56.15 ± 11.27 years, and 58.3% were female. HbA1c decreased significantly from 9.42 ± 1.53% at baseline to 8.21 ± 1.16% at 12 weeks and 7.06 ± 0.69% at 24 weeks (P < 0.001). Significant improvements occurred in FBG and PPBG, triglycerides, low-density lipoprotein cholesterol (LDL-C), total cholesterol (TC), and high-density lipoprotein cholesterol (HDL-C) (all P < 0.001). Liver enzymes and serum creatinine stayed within normal ranges, and body weight remained stable. Dose reduction or discontinuation of concomitant antihyperglycemic therapy occurred in 36.7% of patients; among the 12 insulin-treated patients, insulin was discontinued in eight (66.7%) and down-titrated in 2 (16.7%) (combined, 83.3%). No documented adverse events were identified in the retrospective records.

Conclusion: Saroglitazar was associated with significant improvements in glycemic and lipid parameters without weight gain in non-MASLD T2DM patients ineligible for pioglitazone. Given the uncontrolled retrospective design and concurrent modification of background antihyperglycemic therapy, these findings should be considered hypothesis-generating and warrant prospective evaluation of saroglitazar as an insulin-sensitizing option in this population.

## Introduction

Type 2 diabetes mellitus (T2DM) is a rapidly expanding non-communicable disease burden. The International Diabetes Federation Diabetes Atlas 11^th^ Edition estimates that 589 million adults aged 20-79 years are living with diabetes globally, projected to reach 853 million by 2050 [1]. India has approximately 101 million diabetic cases, representing a major share of the global burden [2]. T2DM is characterized by progressive insulin resistance with relative beta-cell insufficiency, often requiring insulin-sensitizing therapy [3].

Current management of T2DM follows a stepwise, individualized approach, beginning with lifestyle modification and metformin as first-line therapy, with add-on agents selected according to the patients' comorbidities, cardiorenal risk, hypoglycemia risk, and cost, in line with the American Diabetes Association (ADA)/European Association for the Study of Diabetes (EASD) consensus framework. Second- and third-line options include sulfonylureas, dipeptidyl peptidase-4 (DPP-4) inhibitors, sodium-glucose cotransporter-2 (SGLT-2) inhibitors, glucagon-like peptide-1 receptor agonists, insulin, and thiazolidinediones, each targeting distinct components of the 'ominous octet' of T2DM pathophysiology, including insulin resistance, beta-cell dysfunction, and increased hepatic glucose output [3]. Among these, pioglitazone remains the only approved insulin-sensitizing thiazolidinedione; agents that directly target peripheral and hepatic insulin resistance without a thiazolidinedione-class safety burden remain limited, underscoring the need for alternative insulin-sensitizing strategies such as saroglitazar in patients for whom pioglitazone is unsuitable.

Pioglitazone, a selective peroxisome proliferator-activated receptor gamma (PPAR-γ) agonist, is an established insulin sensitizer in T2DM [4]. However, its use is limited by fluid retention, peripheral edema, heart failure exacerbation, fracture risk, and potential bladder cancer risk [5,6]. A meta-analysis reported increased odds of heart failure and fractures with pioglitazone, while the PROactive trial showed higher heart failure hospitalization despite cardiovascular benefits [7-9]. Thus, an unmet need exists for patients requiring insulin sensitization but unable to safely receive pioglitazone.

Saroglitazar, a dual PPAR-α/γ agonist, is approved in India for diabetic dyslipidemia, T2DM as add-on therapy, and metabolic dysfunction-associated steatotic liver disease (MASLD)/metabolic dysfunction-associated steatohepatitis (MASH) indications [10-12]. Its PPAR-α activity improves lipid metabolism, while moderate PPAR-γ activity provides insulin-sensitizing effects with a lower tendency for weight gain and fluid retention than pioglitazone [13,14]. Clinical studies have shown improvements in glycated hemoglobin (HbA1c), triglycerides, low-density lipoprotein cholesterol (LDL-C), high-density lipoprotein cholesterol (HDL-C), and insulin sensitivity with saroglitazar [11,13,14]. However, real-world data in non-MASLD T2DM patients who are ineligible for pioglitazone remain limited. This study therefore evaluated changes in glycemic, lipid, and renal parameters associated with saroglitazar 4 mg once daily over 24 weeks in this population.

## Materials and methods

Study design

This was a single-center, retrospective, real-world study conducted at Indu’s Diabetes Clinic, Mumbai, India. The medical records of adults with T2DM who were initiated on saroglitazar 4 mg once daily as part of routine clinical management between June 2025 and March 2026 were reviewed retrospectively following ethics committee approval. The date of saroglitazar initiation was defined as baseline, and the observation period for each patient was 24 weeks, with follow-up assessments at approximately 12 weeks (Visit 1) and 24 weeks (Visit 2); data recorded beyond 24 weeks were not analyzed. At the time of data extraction, the documented duration of saroglitazar therapy was a median of 11 months (range, six to 24 months).

Study population

Patients were included in the study when the following criteria were met: (i) adults (age ≥18 years) with confirmed T2DM; (ii) patients who had been prescribed saroglitazar 4 mg once daily at the discretion of the treating physician; (iii) patients deemed ineligible for pioglitazone by the treating physician, owing either to an absolute contraindication (heart failure, including New York Heart Association (NYHA) class III/IV) or to clinical unsuitability based on relative precautions (e.g., peripheral edema, osteoporosis/osteopenia, high fracture risk, history of myocardial infarction, or chronic kidney disease (CKD) with edema risk); (iv) no contraindications to saroglitazar; and (v) follow-up data were available for both scheduled post-baseline visits (approximately 12 and 24 weeks). Exclusion criteria were: (i) patients receiving or who had previously received any thiazolidinedione (TZD); (ii) patient data with no primary outcome parameters; or (iii) patients with fewer than two documented post-baseline follow-up visits. Accordingly, the minimum follow-up requirement was two post-baseline visits, and complete data at both Visit 1 and Visit 2 were available for all 60 included patients. All patients had established, long-standing T2DM (mean duration 12.73 ± 5.45 years; median 12 years) and were maintained on their existing background antidiabetic regimen at the discretion of the treating physician at the time saroglitazar was initiated as add-on therapy; saroglitazar was not used as initial or first-line therapy in any patient.

Data collection

Patients were identified from the outpatient and electronic medical records of the study center, and their records were screened against the eligibility criteria by the study investigators. For each eligible patient, data were manually extracted from outpatient case records, electronic medical records, and laboratory reports into a pre-designed, standardized data collection form. The form captured demographic and anthropometric characteristics (age, sex, body weight, and height); duration of T2DM; comorbidities (hypertension, history of myocardial infarction, obesity, CKD, and other documented conditions); saroglitazar dose and duration of therapy; antidiabetic agents and statin use at baseline; modifications of each concomitant antidiabetic agent at baseline, Visit 1, and Visit 2; the clinical and laboratory parameters described under Assessments at each time point; and adverse drug reactions documented during follow-up. All laboratory values were obtained from routine investigations performed as part of standard clinical care at each visit, and no additional investigations were performed for the purpose of this study. Data were de-identified before analysis.

Sample size

As this was a retrospective, real-world analysis, all consecutive eligible patients who received saroglitazar 4 mg during the study period were included (a convenience sample); no a priori sample size calculation was performed, and the 60 patients analyzed represent the entire eligible population identified at the study center. This number reflects the restricted nature of the target population: saroglitazar is commonly prescribed for diabetic dyslipidemia and MASLD, whereas its use for glycemic control in patients without MASLD who are ineligible for pioglitazone represents a small subset of routine clinical practice. A post-hoc power calculation was not performed, as such calculations do not provide meaningful information for a fixed, retrospectively identified cohort. Instead, the adequacy of the sample is reflected in the precision of the estimates, conveyed by the 95% confidence interval (CI) reported for each paired comparison. For the principal glycemic endpoint, the 95% CI for the 24-week reduction in HbA1c (2.04-2.69%) was narrow, and its lower bound exceeded the 0.5% reduction generally regarded as clinically meaningful; the 95% CIs for all glycemic and lipid parameters likewise excluded no change. Consistent with this exploratory design, the findings should be regarded as hypothesis-generating.

Intervention

All patients received saroglitazar 4 mg/day orally, per the treating physician's discretion. Concomitant antidiabetic agents (metformin, sulfonylureas, DPP-4 inhibitors, SGLT-2 inhibitors, and/or insulin) were continued at physician discretion; dose modifications of these agents were documented throughout follow-up.

Assessments

Clinical and biochemical assessments were performed at baseline, 12 weeks (Visit 1), and 24 weeks (Visit 2), evaluating: (a) change in HbA1c (%), fasting blood glucose (FBG, mg/dL), and postprandial blood glucose (PPBG, mg/dL); (b) lipid parameters: triglycerides (TG), LDL-C, total cholesterol (TC), and HDL-C (all mg/dL); (c) liver enzymes: alanine aminotransferase (ALT)/serum glutamic pyruvic transaminase (SGPT) and aspartate aminotransferase (AST)/serum glutamic oxaloacetic transaminase (SGOT) (U/L); (d) serum creatinine (mg/dL); (e) body weight (kg) and body mass index (BMI, kg/m²); and (f) dose modifications of concomitant antihyperglycemic agents. Adverse events voluntarily reported, observed, or elicited within 24 weeks were also recorded.

Statistical analysis

Data were expressed as mean ± standard deviation (SD) for continuous variables and frequency with percentages for categorical variables. The two-tailed paired Student's t-test compared baseline and follow-up values within each group, with a 95% CI computed for each paired comparison. A two-sided *p*-value < 0.05 was considered significant. Each outcome parameter (glycemic, lipid, hepatic, renal, and anthropometric) was pre-specified as an independent efficacy endpoint; no formal adjustment for multiple comparisons was applied. Baseline-to-Visit 1 and baseline-to-Visit 2 comparisons are both reported to show the consistency and time-course of the observed changes.

Ethical considerations

The study was conducted in accordance with the Declaration of Helsinki and applicable regulatory requirements. The study protocol and data collection form were reviewed and approved by the Central Independent Ethics Committee, Pune, India (approval No. CIEC/2026/0196, dated 14 April 2026) prior to data collection. A waiver of informed consent was granted given the retrospective use of de-identified medical record data.

## Results

Baseline patient characteristics

The study included 60 patients with T2DM and documented pioglitazone ineligibility. Mean age was 56.15 ± 11.27 years (range: 35-81 years), and 35 patients (58.3%) were female. Median T2DM duration was 12 years (mean: 12.73 ± 5.45). Mean body weight was 72.92 ± 10.41 kg and mean BMI was 28.14 ± 3.38 kg/m². Comorbidities included obesity (n = 53; 88.3%), hypertension (n = 31; 51.7%), prior myocardial infarction (n = 6; 10.0%), and CKD (n = 5; 8.3%). Metformin was received by 59 patients (98.3%) and a statin by 29 (48.3%) at baseline. Background regimens were frequently complex. Three patients (5.0%) were on metformin monotherapy, six (10.0%) on a two-drug regimen, 20 (33.3%) on a three-drug regimen, and 31 (51.7%) on ≥4 concomitant agents before saroglitazar was added. Concomitant agents included sulfonylureas, DPP-4 inhibitors, SGLT-2 inhibitors, and/or insulin; no patient received any TZD. Pioglitazone ineligibility was determined by the treating physician at the time of clinical assessment; however, the specific reason for ineligibility was not captured as a discrete variable in the structured data collection form, and a patient-level breakdown by reason could therefore not be derived retrospectively. Among the recorded comorbidities, those relevant to pioglitazone use were history of myocardial infarction (n = 6; 10.0%), CKD (n = 5; 8.3%), osteoporosis (n = 2; 3.3%), and heart failure with preserved ejection fraction (n = 1; 1.7%); overall, 12 patients (20.0%) had at least one of these conditions documented. Apart from heart failure, these conditions represent relative precautions rather than absolute contraindications to pioglitazone. Baseline characteristics are summarized in Table 1.

**Table 1:** Baseline patient characteristics (n = 60) T2DM: type 2 diabetes mellitus; BMI: body mass index

Characteristic	Value, n (%) or Mean ± SD
Age (years)	56.15 ± 11.27
Sex, male	25 (41.7%)
Sex, female	35 (58.3%)
Duration of T2DM (years)	12 (median); 12.73 ± 5.45 (mean)
Body weight (kg)	72.92 ± 10.41
BMI (kg/m²)	28.14 ± 3.38
Obesity	53 (88.3%)
Hypertension	31 (51.7%)
History of myocardial infarction	6 (10.0%)
Chronic kidney disease	5 (8.3%)
Metformin use	59 (98.3%)
Statin use	29 (48.3%)

Glycemic outcomes

HbA1c declined significantly from a baseline mean of 9.42 ± 1.53% to 8.21 ± 1.16% at 12 weeks and further to 7.06 ± 0.69% at 24 weeks (absolute reduction: 2.36%; 95% CI: 2.044-2.693; t = 14.5985; *p* < 0.001), a clinically meaningful improvement with the 24-week mean approaching recommended targets for most patients.

FBG decreased from 193.73 ± 61.45 mg/dL at baseline to 146.93 ± 35.16 mg/dL at Visit 1 and 112.03 ± 22.69 mg/dL at Visit 2 (reduction: 81.70 mg/dL; 95% CI: 67.20-96.20; t = 11.2783; *p* < 0.001). PPBG declined from 266.63 ± 86.65 mg/dL at baseline to 184.41 ± 43.23 mg/dL at Visit 1 and 150.98 ± 26.32 mg/dL at Visit 2 (reduction: 115.65 mg/dL; 95% CI: 94.20-137.10; t = 10.7887; *p* < 0.001). Glycemic outcomes are presented in Table 2.

**Table 2:** Changes in glycemic parameters from baseline to follow-up visits (n = 60) Data are presented as mean ± SD. *P* < 0.05 is considered statistically significant. Paired Student's t-test was used for all comparisons. HbA1c: glycated hemoglobin; FBG: fasting blood glucose; PPBG: postprandial blood glucose; CI: confidence interval; SD: standard deviation.

Parameter	Baseline	Visit 1 (12 Weeks)	Visit 2 (24 Weeks)	95% CI	t-value	*p*-value
HbA1c (%)	9.42 ± 1.53	8.21 ± 1.16	7.06 ± 0.69	2.044–2.693	14.5985	*p* < 0.001
FBG (mg/dL)	193.73 ± 61.45	146.93 ± 35.16	112.03 ± 22.69	67.20–96.20	11.2783	*p* < 0.001
PPBG (mg/dL)	266.63 ± 86.65	184.41 ± 43.23	150.98 ± 26.32	94.20–137.10	10.7887	*p* < 0.001

Lipid, hepatic, renal, and anthropometric outcomes

All lipid parameters improved significantly at 24 weeks. Triglycerides declined from 237.86 ± 163.13 mg/dL to 108.16 ± 43.25 mg/dL (reduction: 54.6%; *p* < 0.001). LDL-C decreased from 105.47 ± 42.52 to 69.03 ± 22.60 mg/dL, and TC from 177.59 ± 50.05 to 131.10 ± 29.06 mg/dL (both *p* < 0.001). HDL-C increased from 38.47 ± 7.92 to 44.37 ± 9.09 mg/dL (*p* < 0.001).

Baseline liver enzymes were within normal limits (ALT: 35.33 ± 18.99 U/L; AST: 31.47 ± 14.09 U/L) and remained so throughout. ALT further decreased to 20.59 ± 7.64 U/L and AST to 20.45 ± 5.59 U/L at 24 weeks (both *p* < 0.001).

Body weight remained stable (baseline: 72.92 ± 10.41 kg; Visit 2: 72.94 ± 10.20 kg; *p* = 0.8539), as did BMI (28.14 ± 3.38 vs. 28.15 ± 3.28 kg/m²; *p* = 0.8524). Changes in lipid, liver enzyme, renal, and anthropometric parameters are presented in Table 3.

**Table 3:** Changes in lipid, liver enzyme, renal, and anthropometric parameters (n = 60) For HDL-C, the positive CI indicates an increase from baseline. Data are presented as mean ± SD. Paired Student's t-test was used. ALT: alanine aminotransferase; AST: aspartate aminotransferase; BMI: body mass index; CI: confidence interval; HDL-C: high-density lipoprotein cholesterol; LDL-C: low-density lipoprotein cholesterol; SD: standard deviation; SGOT: serum glutamic oxaloacetic transaminase; SGPT: serum glutamic pyruvic transaminase; TC: total cholesterol; TG: triglycerides.

Parameter	Baseline	Visit 1 (12 Weeks)	Visit 2 (24 Weeks)	95% CI	t-value	*p*-value
Triglycerides (mg/dL)	237.86 ± 163.13	171.72 ± 149.98	108.16 ± 43.25	91.96–167.43	6.8798	*p* < 0.001
LDL-C (mg/dL)	105.47 ± 42.52	80.67 ± 25.62	69.03 ± 22.60	27.28–45.60	7.9615	*p* < 0.001
Total cholesterol (mg/dL)	177.59 ± 50.05	146.49 ± 34.21	131.10 ± 29.06	35.91–57.07	8.7981	*p* < 0.001
HDL-C (mg/dL)	38.47 ± 7.92	40.74 ± 8.04	44.37 ± 9.09	-7.978 to -3.822	5.6828	*p* < 0.001
ALT/SGPT (U/L)	35.33 ± 18.99	26.61 ± 12.35	20.59 ± 7.64	11.047–18.437	7.9867	*p* < 0.001
AST/SGOT (U/L)	31.47 ± 14.09	25.30 ± 10.19	20.45 ± 5.59	8.120–13.911	7.6146	*p* < 0.001
Serum creatinine (mg/dL)	0.88 ± 0.26	0.84 ± 0.24	0.78 ± 0.24	0.0681–0.1274	6.6105	*p* < 0.001
Body weight (kg)	72.92 ± 10.41	73.28 ± 10.43	72.94 ± 10.20	−0.276 to 0.229	0.1849	0.8539
BMI (kg/m²)	28.14 ± 3.38	—	28.15 ± 3.28	−0.108 to 0.089	—	0.8524

The overall changes in glycemic and lipid parameters from baseline to 24 weeks are illustrated in Figure 1.

**Figure 1:**
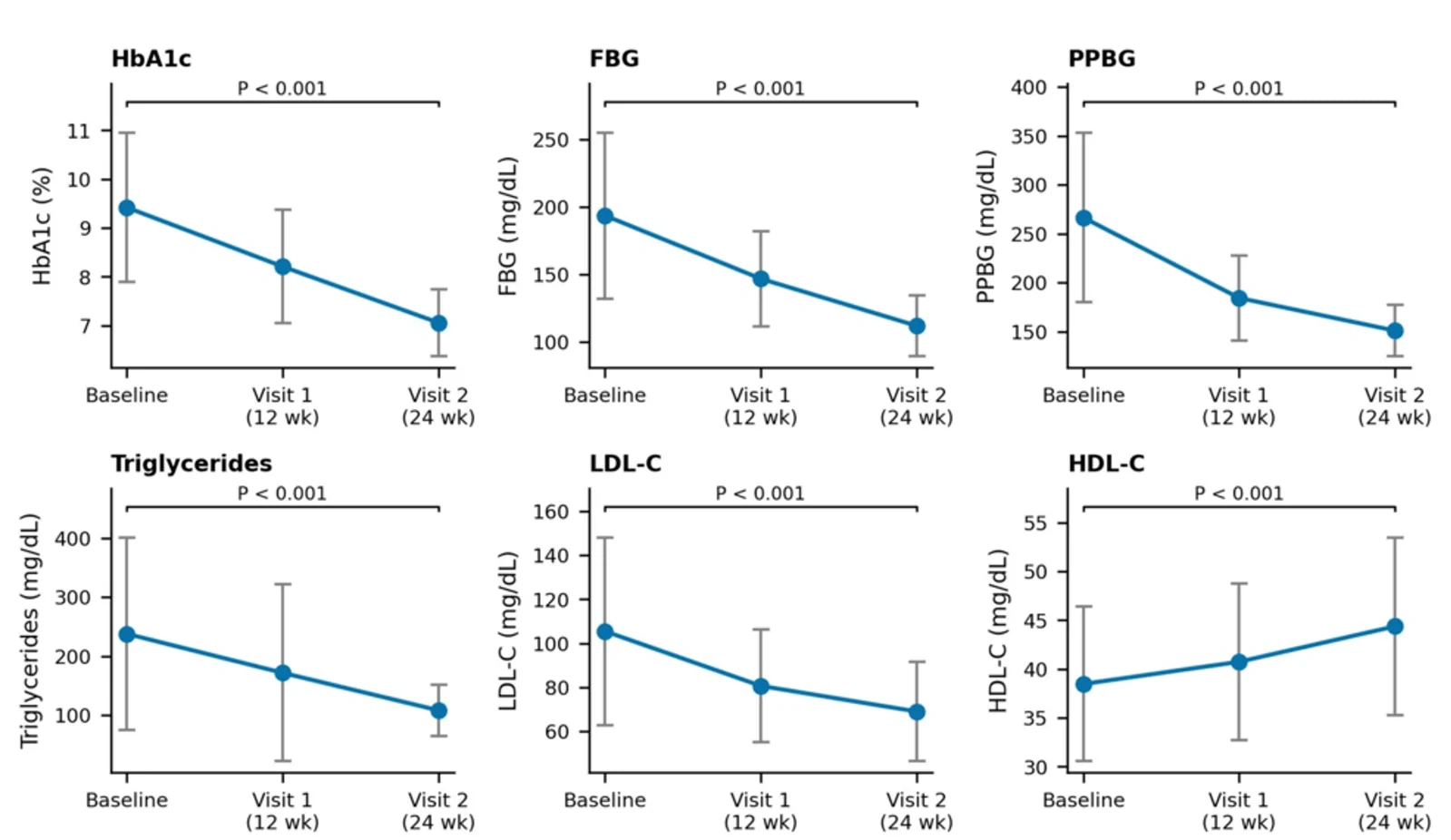
Change in glycemic and lipid parameters from baseline to 24 weeks Data are presented as mean ± SD. *P*-values are from a paired Student’s t-test, baseline vs. Visit 2 (24 weeks); all within-group comparisons (baseline vs. Visit 1 and baseline vs. Visit 2) were statistically significant at p < 0.001 for every parameter shown (Tables 2, 3). No adjustment for multiple comparisons was applied, as each parameter was pre-specified and analyzed as an independent efficacy endpoint. HbA1C: glycated hemoglobin; FBG: fasting blood glucose; PPBG: postprandial blood glucose; LDL-C: low-density lipoprotein cholesterol; HDL-C: high-density lipoprotein cholesterol

Concomitant antidiabetic therapy modifications

At baseline, 51 patients (85.0%) were on DPP-4 inhibitors, 38 (63.3%) on sulfonylureas, 23 (38.3%) on SGLT-2 inhibitors, and 12 (20.0%) on insulin. Over 24 weeks, dose reductions or discontinuations were made at physician discretion as glycemic control improved. Of 38 patients on sulfonylureas, 13 (34.2%) required dose reduction and five (13.2%) discontinued by 24 weeks (total modifications: 18/38; 47.4%). Of 12 patients on insulin, eight (66.7%) discontinued insulin completely (insulin cessation) and two (16.7%) continued insulin at a reduced dose (insulin dose reduction); the remaining two (16.7%) continued insulin unchanged. The combined outcome of insulin cessation or dose reduction was therefore observed in 10 of 12 insulin-treated patients (83.3%). Overall, 22 of 60 patients (36.7%) had at least one dose reduction or discontinuation of a concomitant antihyperglycemic agent. At baseline, sulfonylurea-treated (n = 38) and insulin-treated (n = 12) patients had marked hyperglycemia (mean HbA1c 9.72 ± 1.54% and 9.50 ± 1.07%, respectively), indicating that de-escalation occurred in patients with poorly controlled rather than pre-existing mild disease; all adjustments were made with continued glucose monitoring to mitigate hypoglycemia risk.

Insulin-Treated Subgroup

At baseline, six of the 12 insulin-treated patients (50.0%) were receiving basal insulin (insulin glargine, n = 5; insulin degludec, n = 1) and four (33.3%) premixed insulin; the insulin regimen was not documented for two patients (16.7%), and none was receiving a basal-bolus regimen. All 12 patients were concomitantly receiving metformin and a sulfonylurea. Among the nine patients with a documented dose, the mean total daily insulin dose was 15.8 ± 8.3 international units (IU)/day (median 14 IU/day; range 6-36), corresponding to 0.24 ± 0.13 IU/kg/day. Mean baseline HbA1c in this subgroup was 9.50 ± 1.07%. No protocol-defined de-escalation algorithm or glycemic threshold was applied, and the specific clinical or glycemic criteria prompting de-escalation were not captured in the medical records; insulin was down-titrated or discontinued at the treating physician's discretion as part of routine clinical care. Among the nine patients with documented insulin cessation or dose reduction, de-escalation was first recorded at Visit 1 (approximately 12 weeks), at which point mean HbA1c was 8.40 ± 0.77% and mean FBG was 152.9 ± 30.6 mg/dL. The two dose reductions comprised a switch from premixed insulin (14 IU/day) to insulin glargine (8 IU/day) and a down-titration of insulin glargine from 14 to 8 IU/day. In one patient classified as unchanged, a transient dose reduction at Visit 1 had reverted to the baseline dose by Visit 2. Given the small size of this subgroup (n = 12), these observations are descriptive and hypothesis-generating.

Safety outcomes

No documented adverse events were identified in the retrospective records during the 24-week follow-up, including among patients with CKD, prior myocardial infarction, or heart failure. Specifically, no drug-induced hepatotoxicity, symptomatic hypoglycemia, peripheral edema, weight gain, or other adverse drug reaction was documented. Because safety data were derived from passive retrospective chart review, under-recording of mild or transient events cannot be excluded.

## Discussion

In this retrospective cohort, treatment with saroglitazar 4 mg once daily was associated with significant and sustained improvements in glycemic control and diabetic dyslipidemia over 24 weeks, without weight gain or documented adverse events.

The observed HbA1c reduction of 2.36 percentage points, from 9.42% at baseline to 7.06% at 24 weeks, represents a substantial glycemic response in this real-world cohort. These findings are consistent with the pivotal PRESS XII trial, a 56-week, randomized, double-blind phase 3 study in 1,155 patients with T2DM, which reported an approximately 1.47% reduction in HbA1c with saroglitazar 4 mg at 24 weeks, together with significant improvements in triglycerides and LDL-C [11]. The insulin-sensitizing effects of saroglitazar are multifactorial. Through moderate PPAR-γ agonism, saroglitazar enhances adiponectin expression and peripheral glucose uptake, while PPAR-α activation promotes hepatic fatty-acid oxidation, reduces hepatic triglyceride accumulation, and attenuates lipotoxicity [15]. The relatively high baseline HbA1c in the present cohort (9.42%) may have contributed to the greater absolute reduction observed, consistent with evidence that glucose-lowering efficacy is influenced by baseline glycemic status; concurrent physician-directed changes to background therapy and the absence of a control group may also have contributed, and the magnitude of change should therefore be interpreted with caution [16]. A recent systematic review and meta-analysis of randomized controlled trials similarly demonstrated significant reductions in HbA1c and fasting glucose with saroglitazar, further supporting the glycemic findings of the present study [17]. These mechanistic and clinical findings are further supported by a randomized, double-blind, placebo-controlled trial using hyperinsulinemic-euglycemic clamp methodology, which demonstrated significant improvement in insulin sensitivity with saroglitazar, accompanied by an increase in the homeostatic model assessment of β-cell function (HOMA-β), suggesting beneficial effects on both insulin sensitivity and beta-cell function [18]. Collectively, these findings are consistent with the established pharmacological effects of saroglitazar and extend the available evidence to patients with T2DM who cannot receive pioglitazone.

The lipid-modifying effects-particularly the 54.6% triglyceride reduction (237.86 to 108.16 mg/dL) and significant improvements in LDL-C, TC, and HDL-C-are consistent with saroglitazar's PPAR-α-mediated mechanism and the PRESS trial data [13]. Atherogenic dyslipidemia in diabetes, characterized by hypertriglyceridemia, low HDL-C, and elevated small dense LDL-C, is a major cardiovascular risk factor that persists even after LDL-C lowering with statins alone [19]. The magnitude of triglyceride reduction here (>50%) is clinically significant and may translate into meaningful cardiovascular risk reduction, particularly since 51.7% of patients had hypertension and 10.0% had prior myocardial infarction. A large-scale real-world evidence study in Indian metabolic disease patients similarly reported significant reductions in TG (−55.41 mg/dL) and LDL-C alongside hepatic improvements, corroborating these findings [20]. These findings are further supported by a prospective observational real-world study of saroglitazar in patients with diabetic dyslipidemia, which demonstrated improvements in lipid and hepatic parameters [21].

It is important to note that these lipid improvements occurred on a background of continued, physician-directed statin therapy in the majority of patients, and are not attributable to saroglitazar in isolation. Consistent with its PPAR-α-predominant mechanism, saroglitazar principally lowers triglycerides by upregulating lipoprotein lipase activity and enhancing hepatic fatty-acid oxidation. In contrast, its effect on LDL-C is comparatively modest and is thought to occur indirectly, through a shift from small, dense, atherogenic LDL particles toward larger, more buoyant particles as hypertriglyceridemia improves, rather than through direct LDL-receptor upregulation [13,18]. The concurrent LDL-C and total cholesterol reduction observed here therefore most plausibly reflects the combined effect of background statin therapy and saroglitazar-mediated triglyceride lowering, rather than a direct LDL-lowering action of saroglitazar alone.

Importantly, baseline liver enzymes were within normal limits in this non-MASLD cohort (ALT: 35.33 ± 18.99 U/L; AST: 31.47 ± 14.09 U/L), and no elevation was documented at any time point during the 24-week follow-up, supporting a favorable hepatic tolerability profile in this cohort. The further statistically significant decreases in ALT (to 20.59 U/L) and AST (to 20.45 U/L) at 24 weeks are consistent with saroglitazar's PPAR-α-mediated attenuation of hepatic lipotoxicity and de novo lipogenesis, even in the absence of MASLD [22,23]. While hepatoprotective benefits have been well established in MASH settings [12], the present findings suggest that saroglitazar's favorable hepatic metabolic profile extends to non-MASLD patients, likely through improved insulin sensitivity reducing hyperinsulinemia-driven hepatic fat accumulation. Preclinical data from animal models of non-alcoholic steatohepatitis have further confirmed saroglitazar's transcriptomic PPAR-target activation and anti-inflammatory hepatic effects [24].

The reduction in serum creatinine from 0.88 to 0.78 mg/dL (P < 0.001) was small in absolute magnitude, and values remained within the normal range throughout. Although improved glycemic control, reduced glomerular hyperfiltration, and insulin sensitization could theoretically influence renal hemodynamics [25]. However, in the absence of urinary albumin-to-creatinine ratio (UACR) and estimated glomerular filtration rate (eGFR) trajectory data, and given the retrospective design, this change is insufficient to infer a nephroprotective effect; it may reflect assay or biological variability, changes in hydration status or muscle mass, or concomitant medication adjustments. The more pertinent observation is the absence of any rise in serum creatinine during saroglitazar therapy. Any potential renal effect of saroglitazar warrants prospective evaluation with UACR and eGFR, particularly given that 8.3% of the cohort had established CKD.

The weight neutrality observed with saroglitazar (weight change: +0.02 kg; P = 0.854) in the present study contrasts with pioglitazone, which consistently causes 2-4 kg weight gain through fluid retention and adipogenesis [5]. Weight neutrality is particularly desirable in this cohort, given that 88.3% of patients were obese and several had a history of cardiac disease or heart failure. Saroglitazar's predominantly PPAR-α with moderate PPAR-γ agonist activity limits the adipogenic drive responsible for thiazolidinedione-associated weight gain while preserving insulin sensitization [26]. This profile distinguishes saroglitazar from several previously evaluated PPAR agonists that have either failed clinical trials due to safety concerns or demonstrated unfavorable weight effects [26,27].

A clinically noteworthy finding in this study was the frequent down-titration of concomitant antidiabetic therapy during the study period. Of 38 patients on sulfonylureas at baseline, 47.4% required dose reduction or complete discontinuation by 24 weeks; similarly, of 12 insulin-treated patients at baseline, insulin was discontinued in eight (66.7%) and down-titrated in two (16.7%; combined, 83.3%). Overall, 22 of 60 patients (36.7%) had at least one dose reduction or discontinuation of a concomitant antihyperglycemic agent. This pattern is consistent with, but does not establish, an insulin-sensitizing effect mediated by PPAR-γ agonism [15]. Because de-escalation decisions were made at physician discretion and no control group was available, the contribution of saroglitazar cannot be distinguished from that of other factors, such as reinforcement of lifestyle measures, improved treatment adherence, or regression to the mean. These results further highlight the importance of vigilant monitoring of glucose levels and proactive dose adjustments to minimize the risk of hypoglycemia when saroglitazar is added to regimens containing sulfonylureas or insulin.

No documented adverse events were identified in the retrospective records of the 60 patients over 24 weeks, which is consistent with safety data from multiple clinical trials of saroglitazar [11,13]; however, passive retrospective chart review is not designed to capture all adverse events, and this observation should not be interpreted as a definitive safety profile. Notably, unlike fibrates (PPAR-α agonists), saroglitazar was not associated with an increase in serum creatinine, and unlike pioglitazone (a PPAR-γ agonist), no fluid retention or weight gain was documented [5,28]. These observations suggest that saroglitazar may be a suitable therapeutic option for the complex, multimorbid T2DM population represented in this cohort, although confirmation in prospective studies with systematic safety monitoring is required.

The present study addresses T2DM patients requiring insulin sensitization who cannot receive pioglitazone. With the ageing of the T2DM population and the high prevalence of cardiac disease, osteoporosis, and renal impairment in Indian patients with long-standing T2DM, this therapeutic gap is clinically substantial [2,29]. The ADA/EASD consensus guidelines for T2DM management emphasize the need for individualized, patient-centred pharmacotherapy with consideration of comorbidities [30]. The current data suggest saroglitazar may represent a viable insulin-sensitizing alternative in this context, warranting confirmation in controlled studies.

Several limitations of this study warrant acknowledgment. First, the retrospective design without a control group precludes causal inference. Importantly, 22 of 60 patients (36.7%) had their background antihyperglycemic regimens actively modified during follow-up, predominantly through sulfonylurea and insulin down-titration or discontinuation. Because these physician-directed dose adjustments occurred concurrently with saroglitazar therapy, the independent pharmacodynamic effect of saroglitazar is fundamentally confounded by these changes and cannot be isolated from the present data; the observed glycemic and metabolic changes should therefore be interpreted as associations rather than treatment effects. Unmeasured confounders, including lifestyle modification, treatment adherence, and regression to the mean in patients with elevated baseline HbA1c, may also have contributed. Second, the sample size (n = 60) reflects the number of eligible patients rather than a formal calculation and limits the precision of subgroup estimates, and the single-center design limits generalizability to different geographic or demographic settings. Third, insulin sensitivity was not directly measured using gold-standard clamp methodology; glycemic and lipid improvements serve as surrogate markers of insulin sensitization. Fourth, the absence of MASLD exclusion was structural-the non-MASLD designation refers to the indication for saroglitazar (insulin sensitization rather than MASLD treatment), not to the absence of incidental hepatic steatosis in all patients. Fifth, safety data were obtained through passive retrospective chart review, which may under-record mild or transient adverse events. Sixth, renal assessment was limited to serum creatinine, without UACR or eGFR trajectory data. Finally, pioglitazone ineligibility was determined by the treating physician's clinical judgment; the specific reason for ineligibility was not recorded as a discrete variable, and the documented pioglitazone-relevant comorbidities (present in 20.0% of patients) largely represented relative precautions rather than absolute contraindications; the cohort may therefore include patients who could have received pioglitazone, which should be considered when generalizing these findings. Future prospective, randomized controlled trials with direct insulin sensitivity measurement, larger sample sizes, and longer follow-up are warranted to validate the findings.

## Conclusions

In this retrospective real-world cohort, saroglitazar 4 mg once daily was associated with significant improvements in glycemic control and diabetic dyslipidemia, with no elevation in liver enzymes or serum creatinine. Body weight remained stable, and no documented adverse events were identified in the retrospective records over 24 weeks in non-MASLD T2DM patients deemed ineligible for pioglitazone. These real-world findings suggest potential clinical utility of saroglitazar as an insulin-sensitizing option in this underserved patient subgroup. Prospective randomized trials with direct measurement of insulin sensitivity are warranted to confirm these preliminary observations.

## References

[1] (2026). International Diabetes Federation: IDF Diabetes Atlas. https://diabetesatlas.org/resources/idf-diabetes-atlas-2025/.

[2] Anjana RM, Unnikrishnan R, Deepa M (2023). Metabolic non-communicable disease health report of India: the ICMR-INDIAB national cross-sectional study (ICMR-INDIAB-17). Lancet Diabetes Endocrinol.

[3] Defronzo RA (2009). Banting lecture. From the triumvirate to the ominous octet: a new paradigm for the treatment of type 2 diabetes mellitus. Diabetes.

[4] DeFronzo RA, Inzucchi S, Abdul-Ghani M, Nissen SE (2019). Pioglitazone: the forgotten, cost-effective cardioprotective drug for type 2 diabetes. Diab Vasc Dis Res.

[5] Dormandy JA, Charbonnel B, Eckland DJ (2005). Secondary prevention of macrovascular events in patients with type 2 diabetes in the PROactive Study (PROspective pioglitAzone Clinical Trial In macroVascular Events): a randomised controlled trial. Lancet.

[6] Mamtani R, Haynes K, Bilker WB, Vaughn DJ, Strom BL, Glanz K, Lewis JD (2012). Association between longer therapy with thiazolidinediones and risk of bladder cancer: a cohort study. J Natl Cancer Inst.

[7] Sinha B, Ghosal S (2020). Assessing the need for pioglitazone in the treatment of patients with type 2 diabetes: a meta-analysis of its risks and benefits from prospective trials. Sci Rep.

[8] Erdmann E, Dormandy JA, Charbonnel B, Massi-Benedetti M, Moules IK, Skene AM (2007). The effect of pioglitazone on recurrent myocardial infarction in 2,445 patients with type 2 diabetes and previous myocardial infarction: results from the PROactive (PROactive 05) Study. J Am Coll Cardiol.

[9] Erdmann E, Charbonnel B, Wilcox RG (2007). Pioglitazone use and heart failure in patients with type 2 diabetes and preexisting cardiovascular disease: data from the PROactive study (PROactive 08). Diabetes Care.

[10] Joshi SR (2015). Saroglitazar for the treatment of dyslipidemia in diabetic patients. Expert Opin Pharmacother.

[11] Krishnappa M, Patil K, Parmar K (2020). Effect of saroglitazar 2 mg and 4 mg on glycemic control, lipid profile and cardiovascular disease risk in patients with type 2 diabetes mellitus: a 56-week, randomized, double blind, phase 3 study (PRESS XII study). Cardiovasc Diabetol.

[12] Gawrieh S, Noureddin M, Loo N (2021). Saroglitazar, a PPAR-α/γ agonist, for treatment of NAFLD: a randomized controlled double-blind phase 2 trial. Hepatology.

[13] Pai V, Paneerselvam A, Mukhopadhyay S (2014). A multicenter, prospective, randomized, double-blind study to evaluate the safety and efficacy of saroglitazar 2 and 4 mg compared to pioglitazone 45 mg in diabetic dyslipidemia (PRESS V). J Diabetes Sci Technol.

[14] Kaul U, Parmar D, Manjunath K, Shah M, Parmar K, Patil KP, Jaiswal A (2019). New dual peroxisome proliferator activated receptor agonist-saroglitazar in diabetic dyslipidemia and non-alcoholic fatty liver disease: integrated analysis of the real world evidence. Cardiovasc Diabetol.

[15] Jain N, Bhansali S, Kurpad AV (2019). Effect of a dual PPAR α/γ agonist on insulin sensitivity in patients of type 2 diabetes with hypertriglyceridemia: randomized double-blind placebo-controlled trial. Sci Rep.

[16] Bloomgarden ZT, Dodis R, Viscoli CM, Holmboe ES, Inzucchi SE (2006). Lower baseline glycemia reduces apparent oral agent glucose-lowering efficacy: a meta-regression analysis. Diabetes Care.

[17] Simental-Mendía LE, Simental-Mendía M, Barragán-Zuñiga LJ, Arce-Quiñones M (2025). Effect of saroglitazar on glycaemic parameters: a systematic review and meta-analysis of randomized controlled trials. Diabetes Obes Metab.

[18] Jain MR, Giri SR, Bhoi B (2018). Dual PPARα/γ agonist saroglitazar improves liver histopathology and biochemistry in experimental NASH models. Liver Int.

[19] Parhofer KG (2015). Interaction between glucose and lipid metabolism: more than diabetic dyslipidemia. Diabetes Metab J.

[20] Das S, Gupta S, Lathia T (2025). Evaluation of effectiveness and tolerability of saroglitazar in metabolic disease patients of India: a retrospective, observational, electronic medical record-based real-world evidence study. Cureus.

[21] Goyal O, Nohria S, Goyal P, Kaur J, Sharma S, Sood A, Chhina RS (2020). Saroglitazar in patients with non-alcoholic fatty liver disease and diabetic dyslipidemia: a prospective, observational, real world study. Sci Rep.

[22] Francque S, Szabo G, Abdelmalek MF (2021). Nonalcoholic steatohepatitis: the role of peroxisome proliferator-activated receptors. Nat Rev Gastroenterol Hepatol.

[23] Haas JT, Francque S, Staels B (2016). Pathophysiology and mechanisms of nonalcoholic fatty liver disease. Annu Rev Physiol.

[24] Kumar DP, Caffrey R, Marioneaux J (2020). The PPAR α/γ agonist saroglitazar improves insulin resistance and steatohepatitis in a diet induced animal model of nonalcoholic fatty liver disease. Sci Rep.

[25] DeFronzo RA, Reeves WB, Awad AS (2021). Pathophysiology of diabetic kidney disease: impact of SGLT2 inhibitors. Nat Rev Nephrol.

[26] Jani RH, Kansagra K, Jain MR, Patel H (2013). Pharmacokinetics, safety, and tolerability of saroglitazar (ZYH1), a predominantly PPARα agonist with moderate PPARγ agonist activity in healthy human subjects. Clin Drug Investig.

[27] Staels B, Fruchart JC (2005). Therapeutic roles of peroxisome proliferator-activated receptor agonists. Diabetes.

[28] Mychaleckyj JC, Craven T, Nayak U (2012). Reversibility of fenofibrate therapy-induced renal function impairment in ACCORD type 2 diabetic participants. Diabetes Care.

[29] Prabhakaran D, Jeemon P, Roy A (2016). Cardiovascular diseases in India: current epidemiology and future directions. Circulation.

[30] Davies MJ, Aroda VR, Collins BS (2022). Management of hyperglycemia in type 2 diabetes, 2022. A consensus report by the American Diabetes Association (ADA) and the European Association for the Study of Diabetes (EASD). Diabetes Care.

